# Increasing racially and ethnically underrepresented women in medical school through an innovative program

**DOI:** 10.5116/ijme.5918.b789

**Published:** 2017-05-24

**Authors:** Pamela A. Geller, Alexa Bonacquisti, Janine Barber, Lynn H. Yeakel

**Affiliations:** 1 Department of Psychology, Drexel University, Philadelphia, PA, USA; 2 Institute for Women's Health and Leadership, Drexel University College of Medicine, Philadelphia, PA, USA

**Keywords:** Underrepresented in medicine, diversity in medical education, women’s issues in medical education, medical education programming

## Introduction

In 2014, women comprised nearly half of medical school graduates;^1^ yet, the proportion of female medical students from underrepresented racial-ethnic groups remains low. The Association of American Medical Colleges, recognizing this disparity, introduced the term “underrepresented in medicine” (URM) to refer to those groups that are underrepresented in the medical profession relative to their numbers in the general population.^2^ The United States (US) has experienced a continual increase in diverse populations, but the number of URM physicians has not risen to match this pace.^3^ At the same time, the persistent shortage of primary care physicians, particularly those in family medicine who serve high-need populations,^4^ is a well-documented phenomenon.^5^

The past fifty years have witnessed growth in medical education and practice among URM women; however, there remain significant improvements to be made in terms of permitting access and engagement for these women, specifically related to the debt associated with financing their education and the provision of available mentors who can assist in the career development process. Improvements in this area can offer significant benefits to diverse communities, as a large proportion of underrepresented women who graduate from medical school plan to specialize in primary care and locate their practice in underserved areas. This underscores the need to make systems-level improvements and develop innovative programming to recruit, enroll, and retain minority women in medical education.

One such approach is the Woman One Award and Scholarship Fund program, developed by the Drexel University College of Medicine’s (DUCOM) Institute for Women’s Health and Leadership (IWHL) in Philadelphia, Pennsylvania. Woman One aims to effectively address the primary financial barriers faced by minority women in the pursuit of medical education, thereby increasing access, improving retention, and facilitating their goal of becoming physicians who may practice in underserved communities.

### The Woman One scholarship program

The IWHL, created to address women’s health needs and promote medical education, research, and leadership development for women in medicine and science, introduced the Woman One Scholarship in 2003 as a means for recruiting URM women to DUCOM. DUCOM constitutes the medical institution that was established following the merger between the historic Hahnemann University and Medical College of Pennsylvania (formerly the Woman’s Medical College of Pennsylvania).

The Woman One Award and Scholarship Fund Program was designed with a dual purpose: 1) honor annually an outstanding woman for her humanitarian contributions and exceptional leadership, and 2) raise funds to increase access and retention for talented URM women pursuing medical education with commitment to practice in an underserved area. The program, currently in its 13th year, offers tuition support for four years of medical school. As of 2015, there are 29 Woman One Scholars, 18 of whom have already graduated from DUCOM. The Woman One Scholarship Program is funded by corporate, foundation, and individual donations made to the annual event honoring a woman leader.

### Opportunities for Woman One scholars

The most basic component of the Woman One Scholarship is financial support, totaling $100,000 (as of 2015) for four years of medical school. Scholars also benefit from additional opportunities, such as participation in volunteer activities with medically underserved populations, concurrent enrollment in joint degree programs, research activities, and community outreach and service. In addition, Scholars can participate in one-to-one mentorship programs with faculty to enhance their careers and discuss issues of professional development. Because Woman One program leaders, mentors, and Woman One Award honorees in particular, have strong professional connections in the Philadelphia community, Scholars have opportunities to meet community, government, and civic leaders, as well as prominent figures in the medical field.

### Woman One scholarship program evaluation

Woman One Scholarship Program leaders identify the program goals as follows: “The success of the Woman One Program can be measured in several ways: first and foremost, by the hundreds of thousands of patients in medically-underserved areas who will benefit from the medical care they receive from these talented and committed physicians; second, by the contributions these culturally-diverse students make to their educational institution and classmates; third, by the increased opportunities for minority women to fulfil their dreams of becoming doctors”. Through the results of a structured survey, we have documented and described the success of this program and also provide a model to inform the development of similar programs at other institutions. Survey results showed that a majority of Woman One Scholars intended to pursue primary care or related specialties and all planned to practice in an underserved area. In addition, the survey responses demonstrated that the Woman One program serves to combat barriers for ethnic-racial minorities in medicine by reducing debt and providing additional opportunities such as mentoring, which can reduce gender-related barriers to career advancement.^6^  

## Conclusions

The Woman One Scholarship Program, developed and implemented by the IWHL at DUCOM, is an innovative model with a dual function--both to honor a woman of exceptional leadership and humanitarian accomplishments, and to support the recruitment and retention of talented URM women in medical school. Through financial assistance and targeted mentorship, Woman One aims to reduce the barriers faced by URM women in the pursuit of medical education, thereby increasing access, improving retention, and allowing URM women to reach their goal of becoming physicians who may practice in underserved areas, thereby enhancing access to medical care for many underrepresented groups.

The Woman One Program has implications for diversity among physicians, benefitting both medical education and clinical practice, especially for the treatment of diverse patient groups in multicultural, underserved communities. However, barriers to matriculation exist among racial and ethnic minorities in medical education, including concern about debt and financing their education, as well as the lack of diversity in mentors and faculty.^7^ Surmounting these barriers to recruiting and retaining more URM students, particularly women, in medical school remains an important endeavor.

Despite the influx of women enrolling in medical school in recent years, there still remains an inequitable distribution of URM students relative to their non-minority counterparts. Estimates indicated that fewer than ten percent of US physicians are underrepresented minorities.^3^ Furthermore, diverse women are particularly underrepresented, making efforts to diversify women in medicine sorely needed.^8^ Results from this survey demonstrate that a targeted recruitment and retention strategy such as Woman One can address this inequity. The Woman One Scholarship promotes medical practice in underserved areas, thereby increasing access to quality medical care for a diverse range of patient populations. The Woman One Scholarship exists as a sustainable, innovative model to accomplish these goals, with larger implications for the medical community. This program serves as a model for others in medical education who wish to pursue the targeted goals of increasing diversity among physicians and enhancing access to care among underserved populations.

### Acknowledgements

The authors wish to thank the Woman One Scholars who participated in this survey. They also wish to acknowledge Efrat Eichenbaum, PhD and Mitra Khaksari, MS for their contributions to the development of the survey and data collection.

### Conflict of Interest

PAG and AB have no conflicts of interest to report. JB is the program manager, and LHY is the Director and Betty A. Cohen Chair in Women’s Health, for the Institute for Women’s Health and Leadership, which raises funds to support the Woman One Scholars Program.  
